# Self-management of type 2 diabetes in gulf cooperation council countries: A systematic review

**DOI:** 10.1371/journal.pone.0189160

**Published:** 2017-12-12

**Authors:** Thamer Al Slamah, Barbara I. Nicholl, Fatima Y. Alslail, Craig A. Melville

**Affiliations:** 1 General Practice and Primary Care, Institute of Health and Wellbeing College of Medicine, Veterinary and Life Science, University of Glasgow, Glasgow, United Kingdom; 2 Director of the National Diabetes Control and Prevention Program, Ministry of Health, Riyadh, Kingdom of Saudi Arabia; 3 Mental Health and Wellbeing, Institute of Health and Wellbeing, College of Medicine, Veterinary and Life Science, University of Glasgow, Glasgow, United Kingdom; Weill Cornell Medical College in Qatar, QATAR

## Abstract

**Aims:**

This study aimed to systematically review intervention studies on self-management of type 2 diabetes in Gulf Cooperation Council (GCC) countries to determine the most effective self-management strategies for individuals with type 2 diabetes in this region.

**Methods:**

A search strategy was developed using multiple databases: Medline and Embase (via Ovid), CINAHL (via EBSCO), and PubMed. Study and intervention characteristics, intervention structure, content, cultural adaptation, and outcomes were extracted from the included studies. To be included in the review the studies should have met the following criteria: have examined the effectiveness of at least one intervention involving a type 2 DSME programme, have involved participants over 18 years old diagnosed with type 2 diabetes, have taken place to in a GCC country, have a study design that was observational, quasi-experimental or controlled, have reported at least one individual and have a quantitative outcome. A narrative data synthesis was used to describe the studies and comment on their methodological quality.

**Results:**

Of the 737 retrieved papers, only eight met the inclusion criteria. Only one study was a randomised controlled trial. A statistically significant improvement in HbA1c was reported in five of the eight studies. There was a significant improvement in physical activity levels as reported in four of the eight studies. Only three studies referred to aspects of cultural design or adaptation of the intervention implemented.

**Conclusions:**

Self-management interventions may have a positive impact on HbA1 levels in patients with type 2 diabetes in the GCC area. A greater emphasis placed on culturally appropriate self-management programmes may improve the effectiveness of self-management interventions for adults with type 2 diabetes in the GCC.

## Introduction

Diabetes attracts significant attention globally due to its rapidly increasing prevalence and high costs for individuals, and society in general [1]. The International Diabetes Federation estimated that, worldwide, there were 410 million people living with diabetes, around which 90% have a diagnosis of type 2 diabetes [2]. A stated aim of the World Health Organisation is to increase levels of awareness of the global burden and consequences of diabetes, with a particular focus on developing countries [3].

The number of people with type 2 diabetes in the countries of the Gulf Cooperation Council (GCC)—the Kingdom of Saudi Arabia (KSA), Kuwait, Qatar, Oman, Kingdom of Bahrain, and United Arab Emirates (UAE)—has dramatically increased in the past two decades [4], and is expected to increase by 96.3% by 2035 [2]. In 2015, the estimated prevalence of diabetes in adults (20–79 years) in each of the GCC countries was higher than the global prevalence of 8.8% [2]. In KSA, it was 17.6%; Kuwait, 14.3%; Qatar, 13.5%; Oman, 9.9%; Kingdom of Bahrain, 15.6%; and UAE, 14.6% [2]. Studies have shown that diabetic control is poor amongst adults with type 2 diabetes living in the GCC countries [5]. As a consequence, there is a disproportionate number of type 2 diabetes complications in GCC countries; for example, 40–70% of diabetes-related foot amputations worldwide are in GCC countries [2].

Diabetes self-management education (DSME) has been shown in meta-analyses to be an effective approach to improving glycaemic control and psychosocial outcomes in adults with type 2 diabetes [6, 7]. DSME has been defined as, "The ongoing process of facilitating the knowledge, skill, and ability necessary for prediabetes and diabetes self-care" [8]. The objectives of DSME are to support policymakers and individuals working in the healthcare sector in their efforts to improve healthcare outcomes and, eventually, the general population's quality of life [8].

In many countries, DSME is considered to be an important part of the first line management of type 2 diabetes. Most DSME programmes were first developed in the United States, and therefore the successful implementation of such programmes in other countries or for different ethnic groups are likely to require some form of cultural adaptation. In fact, cultural adaptation was found to be a factor in the effective implementation of a DSME programme for Mexican Americans and this study demonstrated that cultural adaptation had a positive impact on health outcomes, particularly on HbA1c levels [9]. Additionally, adapting a DSME programme so that it is more culturally appropriate has been shown to have a promising result on dietary behaviour among patients with type 2 diabetes in USA [10]. Most of the evidence supporting the effectiveness of DSME and cultural adaptation comes from studies in the United States of America and other high-income, English-speaking countries. This systematic review examines the evidence for the effectiveness of DSME in adults with type 2 diabetes in GCC countries.

## Methods

This systematic review and its procedures were planned, conducted, and reported according to the Preferred Reporting Items for Systematic Reviews and Meta-Analyses (PRISMA) guidelines [11].

### Search strategy

With support from a medical research librarian, an overall strategy was developed to identify papers relevant to diabetes self-management in GCC countries. Customised searches were devised for the databases Medline and Embase (via Ovid), CINAHL (via EBSCO), and PubMed. The most recent search range available on the database was chosen, which included publications between 1996 and October 2015. Appropriate keywords and Boolean logic were used for the terms ‘diabetes mellitus' OR ‘diabetes complications' and ‘diabetes mellitus' AND ‘self-care'. Full details of the search strategy are provided in S1 File.

To ensure comprehensive identification of potentially relevant studies, manual searches of specialised journals were done for the most recent years 2013–2016. The journals included in the manual searches were the International Journal of Diabetes Care, the Journal of International Diabetes Federation, Diabetes, Diabetes Care, Clinical Diabetes and Diabetes Spectrum. Since the research targeted journals and health organisations relevant to GCC countries, searches were also performed in the Saudi Medical Journal, Omani Medical Journal, Kuwait Medical Journal, Bahrain Medical Bulletin, and Qatar Medical Journal, as well as in publications of the Saudi Diabetes and Endocrine Association, MENA Diabetes Leadership Forum 2010 Dubai, and Ministry of Health Saudi Arabia, with a publication period ranging from 2013 to 2016. Lastly, the reference lists of all publications included in the review, and relevant systematic reviews, were read in detail to identify additional potentially relevant studies.

### Eligibility criteria

Eligible studies had to meet five inclusion criteria:

- Examined the effectiveness of at least one intervention involving a type 2 DSME programme; for which interventions referred to treatments involving elements and activities intended to improve participants' knowledge, skills, and ability to perform self-management activities toward improving their glycaemic control (National Standards of Diabetes Self-Management Education and Support, 2012);
- Participants were diagnosed with type 2 diabetes and aged at least 18 years;
- Studies took place in a GCC country (KSA, Kuwait, Qatar, Oman, Bahrain, and UAE);
- The study design was observational, quasi-experimental or controlled studies. Reported at least one individual and had a quantitative outcome (e.g., glycaemic control, knowledge, adherence to medication, physical activity levels, and quality of life).

Exclusion criteria were: performed in non-GCC countries; non-primary intervention studies; studies included participants with type 1 diabetes; abstract only available; non-English language publications.

### Study selection

Studies from databases were exported to Endnote software to be saved and managed. Duplicate articles were removed. A two-stage process was used to identify papers, records, and publications for inclusion in the systematic review. Two researchers (TASA and CAM) independently screened the titles and abstracts of publications. A consensus discussion took place if there was disagreement about inclusions and exclusions. In the second stage, the same two researchers independently read the full text of the articles and completed inclusion/exclusion checklists for each paper. The disagreement was resolved through a consensus discussion. If the two reviewers could not reach consensus regarding some publications, then a third researcher (BN) was consulted to adjudicate.

### Data extraction and quality assessment

Data extraction was performed independently by the two researchers (TASA and CM), and any disagreements were resolved with the aid of a third researcher (BN).

Study quality was rated using the Standard Quality Assessment Criteria for Evaluating Primary Research Papers tool for quantitative studies [12]. Each study was assessed against 14 criteria-oriented items. If the study met the quality criteria fully it was scored as 2; 1 if it partially met the criteria; and 0 if it did not meet the criteria. For some criteria, "not applicable" (N/A) was the rating given. A total score for each paper was calculated by adding the total score across relevant items and dividing by the total possible score [28 –(Number of N/A x 2)]. The quality assessment tool is provided in S1 Table.

### Data coding frameworks

Guidance published by the Cochrane Collaboration was used to categorise the study design of the included studies [13]. Four frameworks were used to code studies based on content, structure, cultural adaptation, and outcomes.

Coding frameworks were completed independently by two researchers (TASA and CM), and any disagreements were also resolved with the aid of a third researcher (BN).

Several DSME-related frameworks were reviewed to develop a suitable framework to code the content of interventions included in the review. The final framework was developed based on criteria for defining a self-management support intervention, and incorporating aspects of education and knowledge, lifestyle, skills, and support, as defined by Galdas et al. (2015), with additional sub-categories for defining self-management intervention content adapted from Peeples et al. (2007) including problem solving, reducing risk, monitoring, and others. S2 Table provides an explanation for each coding category [14, 15].

Coding of the intervention structure was adapted from Fan and Sidani (2009) and included teaching methods, teaching strategies, a format of delivery used, number of diabetes related topics included, number of sessions, total contact hours, duration of the intervention and whether a booster session was delivered [16].

Coding of cultural adaptation was taken from [17] and included eight components that are considered essential components of the process of adapting interventions to be culturally appropriate (language, persons, metaphors, content, concepts, goals, methods, and context) [17]. This coding framework was used due to the clarity of its dimensions and accompanying description of the elements. The dimensions were developed during structural family therapy for Hispanic groups in the United States, but they are also suitable for evaluating the cultural adaptation or development of interventions in other country settings, including GCC countries.

Intervention outcomes were extracted and coded using a format adapted from Alhyas et al. (2011), including key results regarding glycosylated haemoglobin (Hb1Ac), blood pressure, lipid profile, and weight and body mass index (BMI) [18].

### Data analysis and narrative data synthesis

Since this study involved heterogeneous intervention study designs and only one study was a randomised controlled trial, there was a significant risk of bias in the results. Consequently, a meta-analysis was impossible, and a narrative data synthesis was used to describe the studies, comment on their methodological quality and report outcomes.

## Results

**Fig 1:**
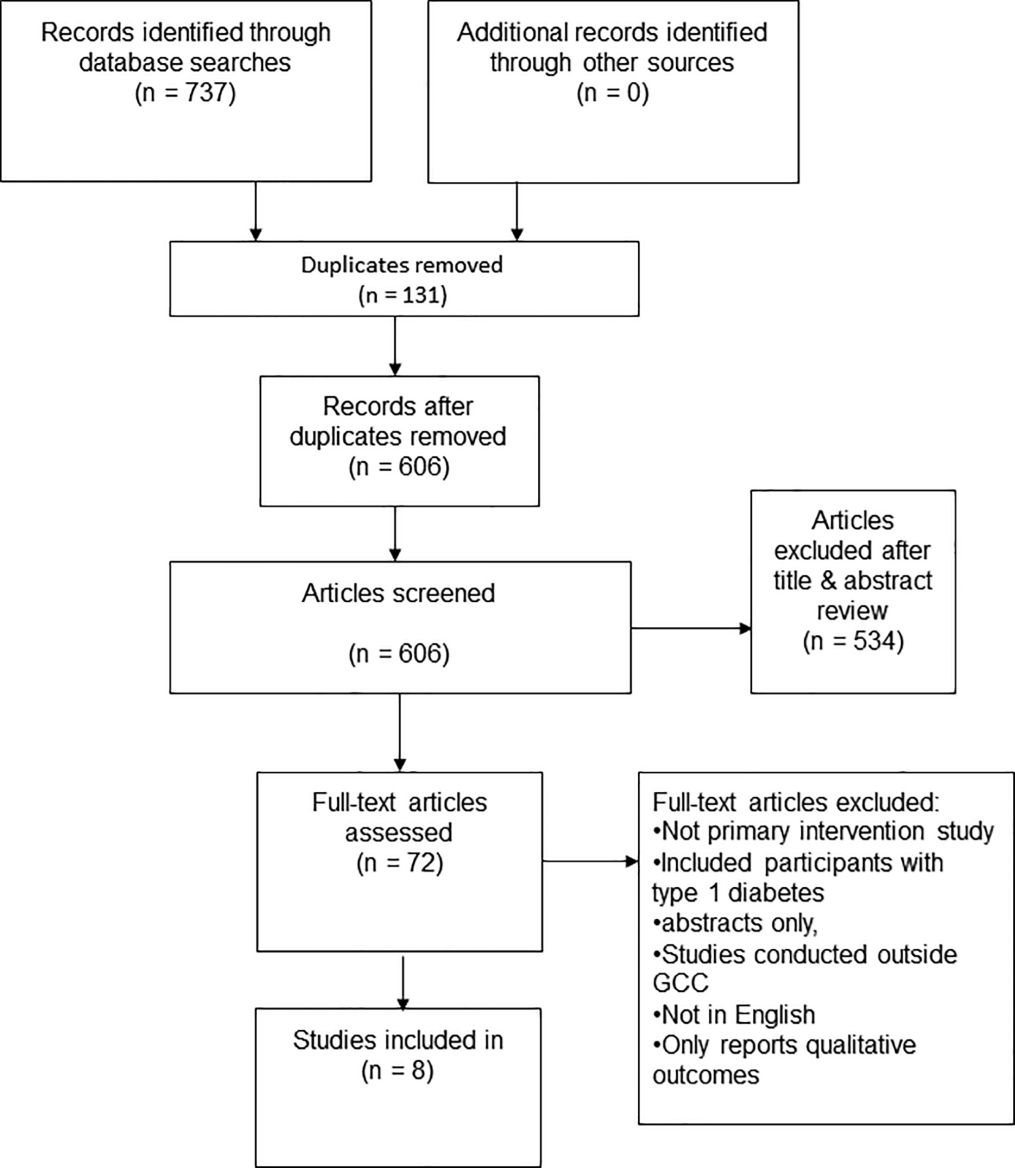
Study flow diagram (PRISMA flow chart).

Fig 1 shows the studies retained at each stage of the study identification and selection process, with eight articles included in the review.

### Study and intervention characteristics

Tables 1 & 2 provide an outline of the eight papers included in the data synthesis and the quality ratings of the studies.

**Table 1:** Characteristics of studies.

			Study characteristics			
Authors	Study setting	Inclusion criteria	Participant traits	Study design	Measures	Assessment quality
Al-Daghri et al. (2014) [21]	Primary care	Not pregnant, without diabetic complications (e.g., renal, neurologic, hepatic, and pulmonary disease), and without acute conditions needing immediate medical attention	T2DM: *N* = 37 (29 F), age 47.69 ± 1.45 years; Pre-diabetes: *N* = 47 (33 F), age 48.85 ± 1.46; non-DMT2: *N* = 66 (51 F), age 39.8 ± 1.44 years)	Controlled before-and-after study	Lipid profile, BMI, blood pressure, serum FBG, serum albumin calcium, phosphate, and vitamin D	0.68
Abduelkarem and Sackville. (2009) [22]	Community based	Taking oral antidiabetic drugs for T2DM, aged <85 years, with normal renal and hepatic function, not pregnant, English or Arabic speaking, and without any cardiovascular disease, chronic disease, or psychological or physical disability	*N* = 59 (32 F), aged 51 ± 11.3 years (range 28–75)	Controlled before-and-after study	General diet, specific diet exercises, foot care, self-testing, body pain, physical functioning, general health, vitality social functioning, and emotional and mental health	0.59
Mohammed et al. (2013) [19]	Community based	Diagnosed with T2DM and registered with primary health care centres and general hospital	Intervention: *N* = 109 (69 F), mean age = 52 ± 8.9 years; Control: *N* = 181 (131 F), aged 55 ± 10.7 years	Randomized controlled trial	HbA1C, FPG, BP, TC, HDL, LDL, TG, BMI, albumin-to-creatinine ratio, and diabetes knowledge, attitude, and practice	0.78
Al-Sinani et al. (2010) [25]	Secondary care	Diagnosed with T2DM	*N* = 98 (49 F), mean age = NA (categorised)	Controlled before-and-after study	HbA1C, FPG, HDL, LDL, TC, TG, BMI, BW, BP, total energy intake per day, carbohydrate, fat, and protein intake/d, energy intake from carbohydrate and fat/d per day, and PA	0.86
Al-Shahrani et al. (2012) [20]	Secondary care	Diagnosed with T2DM, aged >30 years, of Saudi nationality, and completed 5-day diabetic education programme	*N* = 438 (158 F), age = 55.84 ± 10.0 years (range 32–80)	Cohort study	HbA1c, TC, TG, LDL, HDL, BP, BW, and fasting blood sugar	0.77
Al Hayek et al. (2013) [24]	Tertiary care	Aged 18–70 years, diagnosed with T2DM ≥1 year, and of Saudi nationality	*N* = 104 (33 F), age = 57.3 ± 14.4 years	Controlled before-and-after study	HbA1C, HADS, adherence to dietary advice and medication, self-monitoring of blood glucose, and PA	0.59
Alasmary et al. (2013) [23]	Primary care	Diagnosed with T2DM, aged >18 years, and with poorly controlled diabetes	*N* = 41 (24 F), age = 56.2 ± 12.9 years (26–85)	Controlled before-and-after study	HbA1C, FPG, BP, TC, HDL, LDL, TG, and BW	0.77
Omer et al. (2015) [26]	Secondary care	Diagnosed with T2DM, male, aged 40–50 years, and with a BMI = 30.0–34.9 and HBA1C of 9–10%	*N* = 400, age = NR	Controlled before-and-after study	HbA1C, dietary habits, PA, and adherence to medication	0.27

BMI: Body mass index, BP: Blood pressure, BW: Body weight, F: Female, FBG: Fasting blood glucose, HADS: Anxiety and depression, HbA1c: Glycosylated haemoglobin, HDL: High-density lipoprotein, LDL: Low-density lipoprotein, NA: Not available, NR: Not reported, PA: Physical activity, T2DM: Type 2 diabetes mellitus, TC: Total cholesterol, TG: Triglycerides, 0–1: 0 = poor quality, 1 = high quality

**Table 2:** Interventions characteristics.

	Intervention characteristics				
Authors	T2DM intervention	Provider	Offers training	Theoretical model	Duration
Al-Daghri et al. (2014) [21]	Education about lifestyle modifications and need for increased exposure to sunlight; participants asked to self-monitor	Nutritionist physician, nurse, and physical therapists	No	NR	6 months
Abduelkarem and Sackville. (2009) [22]	Weekly reminders on BW, PA, dietary habit, self-testing, foot care, smoking habits, BP, and dyslipidaemia	Pharmacist	No	NR	3 months
Mohammed et al. (2013) [19]	Group health education and counselling sessions	Health educators	Yes	Theory of empowerment, locus of control	NR
Al-Sinani et al. (2010) [25]	Nutrition and lifestyle counselling about diabetes, diet and nutrition, weight management, and exercise	Professional health care team	No	NR	NR
Al-Shahrani et al. (2012) [20]	5-day intensive health education programme	Professional health care team	No	NR	5 days
Al Hayek et al. (2013) [24]	Group health education programme	Nurse diabetes health educators	Yes	NR	6 months
Alasmary et al. (2013) [23]	Multidisciplinary integrated care programme	Family physician, nurse, clinical pharmacy specialist, dietician, health educator, diabetic educator, and social worker	No	NR	6 months
Omer et al. (2015) [26]	Self-monitoring of blood glucose	NR	No	NR	2.5 years

BP: Blood pressure, BW: Body weight, NR: Not reported, PA: Physical activity, T2DM: Type 2 diabetes mellitus.

Of the included studies, one was a randomised controlled trial [19], one was a cohort study [20], and six were controlled before-and-after studies, [21, 22, 23, 24, 25, 26]. Five of the studies were conducted in KSA [20, 21, 23, 24, 26], one study was conducted in the United Arab Emirates [22], one study in Qatar [19], and one study in Oman [25]. None of the studies was conducted in Kuwait or Bahrain.

A total of 1,539 participants were included in the eight studies, with a mean sample size of 139.9 and a range of 37–438. One study reported the age range of participants categorically [25], and one study did not report the age at all [26]. Among the six studies that reported participants' mean age, the combined mean age was 51.5 years with the range of 39–58 years [19, 20, 21, 22, 23, 24].

Six studies measured participant HbA1c as an outcome [19, 20, 23, 24, 25, 26], five studies measured blood pressure [19, 20, 21, 23, 25], five studies measured lipid profile [19, 20, 21, 23, 25], and five studies measured weight or BMI [19, 20, 21, 23, 25]. The duration of intervention was greater than two years in only one study [26].

Only one study explicitly stated the theoretical models used to inform the design of the interventions: the DSME intervention theory of empowerment and locus of control theory [19].

### Content of interventions

Table 3 summarise the intervention content across the eight studies.

**Table 3:** Coding of the content of the DSME interventions used in the eight included studies.

	= Yes, Blank = No													
	Education / Knowledge			Lifestyle				Skills			Support			
Authors	Dietary	Physical Activity Guidance	Other Sources	Healthy Food	Being Active	Monitoring	Taking Medications	Problem Solving	Reducing Risks	Healthy Coping	Monitoring & Feedback	Psychological Interventions	Peer Support	Financial Incentives
Al-Daghri et al. (2014) [21]	•	•	•	•	•	•					•			
Abduelkarem and Sackville. (2009) [22]	•	•	•	•	•	•								
Mohammed et al. (2013) [19]	•	•	•	•	•	•				•		•		
Al-Sinani et al. (2010) [25]	•	•	•	•	•		•							
Al-Shahrani et al. (2012) [20]	•	•	•	•	•	•	•			•				
Al Hayek et al. (2013) [24]	•	•	•	•	•	•	•					•		
Alasmary et al. (2013) [23]	•		•	•		•					•			
Omer et al. (2015) [26]	•	•		•	•	•					•			

All studies except [23] included educational content about physical activity or provided information about active lifestyles in the intervention. Seven of the eight studies included monitoring of blood glucose intervention as part of the content on lifestyle [19, 20, 21, 22, 23, 24, 26], but only three of the studies included content about medication as part of the intervention content [20, 23, 24]. None of the studies included problem-solving skills or skills for reducing risk in the content of their interventions. Furthermore, only two studies incorporated training related to healthy coping skills in their interventions [19, 20], while only three studies involved monitoring and feedback [21, 23, 26].

### Intervention structure

Table 4 summarises the intervention structure in the eight studies.

**Table 4:** Coding of structure of DSME intervention in the eight included studies.

	= Yes, Blank = No																				
	Teaching Method used		Strategies					Format		Number of Diabetes-Related Topics		Number of Sessions			Total Contact Hours			Duration			Delivery of Booster Session
Authors	Didactic	Interactive	Written Material	Online/Web-based	Video	Face-to-Face		One-to-One	Group	Focus on One Topic	One or More Topics	≤5	6–10	>10	≤10	11–20	>20	≤8 weeks	9–24 weeks	>24 weeks	
Al-Daghri et al. (2014) [21]		•				•			•		•										
Abduelkarem and Sackville. (2009) [22]	•		•						•		•								•		
Mohammed et al. (2013) [19]	•	•	•			•			•		•					•				•	
Al-Sinani et al. (2010) [25]	•	•				•		•			•									•	
Al-Shahrani et al. (2012) [20]	•	•	•		•	•			•		•						•	•			
Al Hayek et al. (2013) [24]	•		•		•	•		•	•		•			•						•	
Alasmary et al. (2013) [23]	•	•						•			•			•						•	
Omer et al. (2015) [26]	•					•		•			•		•							•	

Six studies adopted a face-to-face delivery method, and no online or web-based strategies were used in any of the studies. The number of sessions was not reported in three of the studies [21, 22, 25], and only two studies reported the total contact hours involved in their interventions [19, 20]. Lastly, none of the studies offered a booster session following the participants' completion of the formal intervention.

### Intervention cultural adaptation

One of the eight studies described cultural adaptation for the language used in the intervention [22]. This study acknowledged and addressed issues around translation. Another study reported the use of concepts and methods, linking the concepts of empowerment with the practical skills of self-management [19]. One of the eight studies described cultural dimensions of person, content, and context; with regards to the person this study discussed, the encouragement of the patients to share their knowledge, coping with content highlighted special cultural occasions, and context refers to the adaptation of self-management tools to fit the cultural environment [20]. All the remaining studies did not report the use of Bernal's eight dimensions for the development/adaptation of interventions for different cultures [17].

### Intervention outcomes

Tables 5 & 6 shows the key results from the eight studies.

**Table 5:** Outcomes reported glycaemic control and BP Control.

	Measures of Glycaemic Control		Measures of BP Control (mmHg)	
Authors	HbA1c Levels MD (95% CI)	FBG Levels (mM) MD (95% CI)	Systolic BP MD (95% CI)	Diastolic BP MD (95% CI)
Al-Daghri et al. (2014) [21]			0.3 (-0.09, 0.7)	-0.03 (-4.27, 3.67)
Abduelkarem and Sackville. (2009) [22]				
Mohammed et al. (2013) [19]	−0.55 (−0.94, −0.16)	−0.92 (−1.66, −0.18)	0.72 (−2.25, 3.69)	1.30 (−1.85, 4.44)
Al-Sinani et al. (2010) [25]	Male: 0.6 (-0.21, 1.41) Female: 0.1 (-0.75, 0.95)	Male 3.8 (1.94, 5.65) Female 2.4 (0.95, 3.84)	Male -3.2 (-8.51, 2.11) Female -1.1 (-6.87, 4.67)	Male 0.9 (-1.83, 3.63) Female 2.3 (-2.01, 6.61)
Al-Shahrani et al. (2012) [20]	0.91 (0.68, 1.13)	1.81 (1.49, 2.12)	8.19 (6.16, 10.22)	4.37 (3.34, 5.39)
Al Hayek et al. (2013) [24]	Baseline = 8.3 After 6 Months = 7.2			
Alasmary et al. (2013) [23]	1.9 (0.88, 2.91)	3.3 (1.11, 5.48)	1 (-4.41, 6.41)	-0.4 (-3.61, 2.81)
Omer et al. (2015) [26]	Before: SMBG group = 9.5%; Non-SMBG group = 9.3% After 30 Months: SMBG group = 7.8% Non- SMBG group = 8.9%			

MD: Means Difference, BP: Blood Pressure, SBP: Systolic Blood Pressure, DBP: Diastolic Blood Pressure, SD: Standard Deviation, FBG: Fasting Blood Glucose, HbA1c: Glycosylated Haemoglobin, SMBG: Self-Monitoring Blood Glucose, CI: Confidence Intervals.

**Table 6:** Outcomes reported lipid control and other measures.

	Measures of Lipid Control (TC, LDL, HDL, TG)				Other measures	
Authors	TC (mM) MD (95% CI)	LDL (mM) MD (95% CI)	HDL (mM) MD (95% CI)	TG (mM) MD (95% CI)	Weight (Kg) MD (95% CI)	BMI (kg/m2) MD (95% CI)
Al-Daghri et al. (2014) [21]	0.04 (0.38, 0.42)	0.04 (0.37, 0.42)	0.23 (0.22, 0.24)	0.1 (0.08, 0.11)		0.1 (-0.01, 0.2)
Abduelkarem and Sackville. (2009) [22]						
Mohammed et al. (2013) [19]	0.15 (−0.08, 0.37)	0.09 (−0.05, 0.24)	0.16 (0.09, 0.22)	0.05 (−0.03, 0.12)		−1.70 (−2.81, −0.60)
Al-Sinani et al. (2010) [25]	Male -0.1(-3.46, 3.26) Female 0.6 (0.07, 1.12)	Male 0.9 (-0.36, 2.16) Female 0.2 (-0.17, 0.57)	Male 0.1 (-0.11, 0.31) Female 0.2 (-0.39, 0.79)	Male 0.6 (-0.32, 1.52) Female 0.2 (-0.15, 0.55)	Male -2.7 (-7.69, 2.29) Female 4.1 (-0.57, 8.77)	Male -0.3 (-1.88, 1.28) Female 1.7 (-0.81, 3.58)
Al-Shahrani et al. (2012) [20]	0.87 (0.76, 0.97)	0.56 (0.47, 0.64)	-0.04 (-0.08, 0.003)	0.47 (0.36, 0.57)	0.61(-1.18, 2.40)	
Al Hayek et al. (2013) [24]						(Mean) (SD) = (31.063) (4.4)
Alasmary et al. (2013) [23]	0.4 (0.05, 0.85)	0.2 (-0.10, 0.50)	0.1 (0.01, 0.18)	0.6 (-0.29, 1.49)	-1.2 (-7.45, 5.05)	
Omer et al. (2015) [26]						

MD: Means Difference, SD: Standard Deviation, TC: Total Cholesterol, LDL: Low Density Lipoprotein, HDL: High Density Lipoprotein, TG: Triglycerides, BMI: Body Mass Index, CI: Confidence Intervals.

Six of the eight studies reported the effectiveness of their intervention on glycaemic control indicators [19, 20, 23, 24, 25, 26]. Of these, five reported statistically significant positive changes in HbA1c [19, 20, 23, 24, 26], and one study reported no change in HbA1c [25]. Of the five studies that reported blood pressure as an outcome, two reported statistically significant improvements in participant blood pressure [19, 25], but the remaining three studies did not report any change in blood pressure [21, 23, 25]. Four of the eight studies reported significant improvement in physical activity in their results [22, 24, 25, 26]. Among the eight studies, only one study measured patient knowledge and attitude using educational sessions and they observed a statistically significant improvement in this outcome in [19].

## Discussion

This review examined available evidence on the effectiveness of self-management of type 2 diabetes in GCC countries. We found that DSME interventions can have a positive impact on glycaemic control as indicated by blood HbA1c levels. However, there is a need for controlled studies in this area. The studies lacked proper theoretical models which hinders their effectiveness and reliability [27]. Most of the studies focused on education/knowledge and lifestyle and there was a lack of focus on skills and support in the intervention content, despite the fact that both the terms ‘skills’ and ‘support’ were identified as key factors associated with improved quality of life of patients with type 2 diabetes [28]. The studies showed that patients with type 2 diabetes who had received DSME felt more enabled to use their self-management skills; therefore, DSME improved their perceived self-efficacy [29]. A further finding, was that the DSME lacked effective cultural adaptation. This was found to be a hindrance in the effective implementation of the interventions.

Structured DSME programs for patients with newly diagnosed type 2 diabetes can lead to improved belief about illness; resulting in smoking cessation and weight loss [28]. These findings were reported in a multicentre cluster randomised controlled trial by Davies et al, however, they did not observe a significant difference in the HbA1c levels during a 12-month period [28]. DSME appeared to have a positive impact on HbA1c levels in some GCC countries (Saudi Arabia, Qatar and Oman) as observed in this study. This finding agreed with the recent review by Chrvala et al. (2015), conducted in the USA, who also reported that self-management education and support alongside contact time and supportive methods from health providers can positively help patients with type 2 diabetes manage their condition and improved their HbA1c levels [30]. Blood glucose and blood pressure both have an impact on type 2 diabetes and if poorly controlled can result in complications [27]. These findings support the role of continuously self-monitoring levels of these two elements. However, none of the studies included in this review reported long-term follow-up after the intervention, which makes it difficult to assess the long-term effectiveness of their programs. The long-term effect is important to evaluate the effectiveness of an intervention. For instance, a study conducted in the United States and published by Diabetes Prevention Program Research Group (2009) has followed participants over the course of ten years, and found a reduction in diabetes incidence [31]. Thus there is a need to establish a reasonable follow-up period for DSME interventions that allows reliable evaluation of the long-term benefits of such programs.

Coding the content of the DSME interventions revealed that there was a lack of content addressing skills and support within the studies included in this review.

Previous studies highlight the importance of skills training and support in promoting self-management for type 2 diabetes. Having information available to patients regarding self-assessment skills and the support that they can access is associated with a higher degree of self-care behaviours and improved outcomes [32, 29]. However, we found that the studies included in this review lacked this information.

The success of DSME, like any other intervention, requires a clearly formulated theoretical rationale that permits assumptions about the intervention and evaluates these assumptions through its experimental design [33]. A proper theoretical framework should consider the different circumstances of the diabetic patient, such as patient’s demographics, socioeconomic status, lifestyle and nutritional choices, cultural values and traditions, and their access to health provision. Furthermore, the framework should also take into account the patient’s physical health and general mental health and wellbeing [34]. In this review, the included studies did not provide a sufficient consideration of the theoretical rationale of the DSME; therefore, this might compromise their effectiveness. Xu et al. (2008) suggested that the factors which have direct or indirect impact on diabetes self-management include: provider-patient communication, diabetes education and its duration, as well as social support [35]. All of these factors improve patient knowledge leading to self-efficacy [35]. In addition, the improved knowledge creates a positive belief in the intervention plan. The increased self-efficacy, patient self-confidence, and improved knowledge about the disease can result in better self-management of the diabetes. Thus it is necessary that subsequent studies in this area try to develop clear theoretical frameworks encompassing these variables.

The effectiveness of self-management strategies of type 2 diabetes requires that the interventions be tailored to the specific needs of an individual patient in accordance with their personal characteristics [36]. Interventions commonly focus on diet, physical exercise, monitoring of blood glucose, and antidiabetic medications in order to achieve an acceptable glycaemic control. The DSME intervention has to be customised to develop the required skills, attitudes, and abilities to implement self-care within the cultural and social context of each patient. It is widely believed that cultural adaptation is an important aspect of DSME [10, 36]. However, the studies considered in this review revealed that cultural adaptation in DSME is lacking in the GCC countries. Yet, cultural adaptation was highlighted in a study by Brown et al. (2002), which developed an intervention specifically for the Spanish-speaking population that should that it increased participants' knowledge of diabetes [9]. However, cultural adaptation is more than just translation of tools and language. A full translation to allow the DSME to be used effectively by a range of health providers and patient groups requires adaptation of language, understanding and practical application. This shows that there is need for full cultural adaptation of the DSME as an intervention for self-management of diabetes in GCC countries.

### Strengths

This review was conducted in a systematic manner, ensuring all studies related to the research aim were included. We also used a theoretical framework for coding the content of interventions in the included studies. Theoretical frameworks facilitate the comparison of interventions by characterising their content using codes [27].

### Limitations

Despite the strengths of our review, it also has some weaknesses. This review included studies published in the English language only, which may limit its accuracy, as studies that have been reported in Arabic language were excluded. Within the same context, of the eight studies included in this review, only one was a randomised controlled trial and this limits the strength of this review and does not allow us to fully address our second research objective, to determine the most effective self-management strategies for people with type 2 diabetes in GCC. The studies analysed in this work are heterogeneous in their nature, accordingly it is only possible to conduct a narrative analysis. In addition, two studies which both meet the inclusion criteria had significant shortcoming: Al Daghri et al. (2014) [21] had as its primary objective serum vitamin D analysis in diabetes patients and the report by Al Sinani et al. (2010) [25] was based upon a three year gap between intervention and follow up measurements.

### Future research

Considering the limitations highlighted above it is concluded that it is not possible to achieve the stated aim of determining the most effective self-management strategies for individuals with type 2 diabetes in the GCC region by reviewing the published literature. It is, therefore, important to conduct research to identify the most effective forms of intervention in GCC countries. The DSME looks promising, but its effectiveness is unknown at present. This review considered several interventions but was unable to specify the most effective one. Raising awareness and knowledge of diabetes in communities seems to be an attractive area of research in the GCC due to the lack of studies on self-management. Minimal use of theoretical frameworks and cultural adaptation in the studies reviewed are a threat to the effectiveness of DSME interventions. Culturally, the communities in GCC are different from those in developed, western countries where DSME material was first developed, and thus there is a need to develop GCC specific interventions for type 2 diabetes self-management, which is now at epidemic levels. Thus, future work should follow guidance on cultural adaptation to make interventions more effective.

### Conclusion

In conclusion, self-management interventions appear to have a positive impact on type 2 diabetes by decreasing HbA1c levels. The reviewed studies did not always include measures on skills and support in their interventions, which the authors feel are key to improving patient self-efficacy and engagement with self-management of their condition.

## Supporting information

S1 File Search strategy. (DOCX)

S1 Table Quality assessment. (DOCX)

S2 Table Explanation for coding category of intervention content. (DOCX)

S3 Table PRISMA 2009 checklist. (DOCX)
