# Supplementary material for: Self-management of type 2 diabetes in gulf cooperation council countries: A systematic review
Source: PLoS One. 2017 Dec 12;12(12):e0189160. doi: 10.1371/journal.pone.0189160 (PMC5726716; doi:10.1371/journal.pone.0189160)
Supplement: S1 File — (DOCX) [file pone.0189160.s001.docx]

**S 1 File. Search strategy**

Self-Management of Type 2 Diabetes in Gulf Cooperation Council Countries: A Systematic Review

**This research will take into consideration the following aspects:**

1. Clear research questions and research objectives for focused and specific search results.
2. The research will target diabetes type 2, specifically self-management, health education, health promotion, diet and physical activity.
3. The focus of the research (inclusion and exclusion)
4. Define any alternative terminologies, abbreviation list, and other substitutes.
5. Use Boolean logic including “AND, OR”
6. Using multiple sources for the research.
7. Consider studies that were undertaken by the WHO and other relevant organisations.

**Describing electronic database searches**

Using Medline and Embase (via Ovid, respectively, Midline 1996 to October Week 5 2015, Embase 1996 to 2015 Week 45). Using CINAHL (via EBSCOhost) and using PubMed.

1. (Medline) Ovid Search strategy on 25/11/2015

| **Terms** | **Search numbers** |
| --- | --- |
| 1. exp Diabetes Mellitus/ | 214018 |
| 1. exp Diabetes Complications/ | 65918 |
| 1. exp insulin resistance/ | 54285 |
| 1. diabet$.tw,ot. | 294010 |
| 1. (NIDDM or MODY or T2DM or T2D).tw,ot | 13506 |
| 1. impaired glucose toleranc$.tw. | 6735 |
| 1. glucose intoleranc$.tw. | 5118 |
| 1. insulin$ resistanc$.tw. | 45278 |
| 1. 1 OR 2 OR 3 OR 4 OR 5 OR 6 OR 7 OR 8 | 359966 |
| 1. exp Patient education/ | 53005 |
| 1. exp Self Care/ | 31786 |
| 1. exp Patient participation/ | 14826 |
| 1. ((self or symptom$) adj (care or help or manag$ or directed or monitor$ or efficacy or admin$)).tw | 60603 |
| 1. ((health or patient$) adj2 (educat$ or information or promotion)).tw | 71733 |
| 1. 10 OR 11 OR 12 OR 13 OR 14 | 191819 |
| 1. Saudi Arabia OR Kuwait OR Bahrain OR Emirates OR Qatar OR Oman | 11044 |
| 1. 9 AND 15 AND 16 | 90 |

2. (Embase) Ovid Search strategy on 25/11/2015

| **Terms** | **Search numbers** |
| --- | --- |
| 1. exp Diabetes Mellitus/ | 551561 |
| 1. exp Diabetes Complications/ | 551561 |
| 1. exp insulin resistance/ | 85314 |
| 1. diabet$.tw,ot. | 527430 |
| 1. (NIDDM or MODY or T2DM or T2D).tw,ot | 31035 |
| 1. impaired glucose toleranc$.tw. | 11316 |
| 1. glucose intoleranc$.tw. | 8638 |
| 1. insulin$ resistanc$.tw. | 75214 |
| 1. 1 OR 2 OR 3 OR 4 OR 5 OR 6 OR 7 OR 8 | 690141 |
| 1. exp Patient education/ | 75667 |
| 1. exp Self Care/ | 45305 |
| 1. exp Patient participation/ | 16639 |
| 1. ((self or symptom$) adj (care or help or manag$ or directed or monitor$ or efficacy or admin$)).tw | 94780 |
| 1. ((health or patient$) adj2 (educat$ or information or promotion)).tw | 112481 |
| 1. 10 OR 11 OR 12 OR 13 OR 14 | 285196 |
| 1. Saudi Arabia OR Kuwait OR Bahrain OR Emirates OR Qatar OR Oman | 19035 |
| 1. 9 AND 15 AND 16 | 192 |

3. CINAHL (EBSCOhost) Search strategy on 25/11/2015

|  | **Terms** | **Search numbers** |
| --- | --- | --- |
| S1 | (MH "Diabetes Mellitus, Type 2") OR (MH "Diabetes Mellitus") OR (MH "Diabetic Patients") | 58725 |
| S2 | (MH "Insulin Resistance") | 6199 |
| S3 | (MH “Glucose Intolerance) | 1536 |
| S4 | (“non insulin* depend*” OR “noninsulin* depend*” OR “non insulin* depend*” OR “noninsulin* depend*”).ti,ab | 78 |
| S5 | (NIDDM OR MODY OR T2DM OR T2D).ti,ab | 28 |
| S6 | S1 OR S2 OR S3 OR S4 OR S5 | 63150 |
| S7 | (MH "Self Care") | 20896 |
| S8 | (MH "Self Administration") | 1912 |
| S9 | ti self care or ti self help or ti self manag* or ti self directed or ti self monitor* or ti self efficacy or ti self admin* | 11328 |
| S10 | (MH "Consumer Participation") | 10705 |
| S11 | (MH "Patient Education") OR (MH "Health Education") OR (MH "Diabetes Education") | 58825 |
| S12 | (MH "Patient Centered Care") | 14895 |
| S13 | ab self care or ab self help or ab self manag* or ab self directed or ab self monitor* or ab self efficacy or ab self admin* | 938381 |
| S14 | ti health N2 educat* or ti health N2 information or ab health N2 educat* or ab health N2 information | 9380 |
| S15 | ti patient* N2 educat* or ti patient* N2 information or ab patient* N2 educat* or ab patient* N2 information | 5771 |
| S16 | ti patient* participat* or ab patient* participat* or ti consumer* participat* or ab consumer* participat | 820 |
| S17 | (MH “Empowerment”) | 7798 |
| S18 | ti empower* or ab empower* | 3954 |
| S19 | (MH "Saudi Arabia") | 1872 |
| S20 | (MH "Kuwait") | 395 |
| S21 | (MH "Oman") | 212 |
| S22 | (MH "Bahrain") | 183 |
| S23 | (MH "Qatar") | 208 |
| S24 | (MH "United Arab Emirates") | 476 |
| S25 | S7 OR S8 OR S9 OR S10 OR S11 OR S12 OR S13 OR S14 OR S15 OR S16 OR S17 OR S18 | 982629 |
| S26 | S19 OR S20 OR S21 OR S22 OR S23 OR S24 | 3254 |
| S27 | S6 AND S25 AND S26 | 57 |

4. PubMed Search strategy on 25/11/2015

((((((((((((diabetes) OR diabetes mellitus) OR diabetes type2) OR insulin dependent diabetes mellitus) OR glucose intolerance) OR insulin resistance) OR NIDDM) OR T2DM) OR MODY)) AND (((((((((((((self-care) OR self-management) OR self-administration) OR consumer participation) OR patient centred) OR patient participation) OR patient monitor) OR patient manage) OR patient measure) OR patient education) OR patient Diet) OR patient activity) OR patient adjust))) AND ((((((Saudi Arabia) OR Qatar) OR Oman) OR Kuwait) OR Bahrain) OR emirates)

Items found: 398

**Describing journal hand searches (2013-2015)**

1. International Journal of Diabetes Care
2. Diabetes Research and Clinical Practice
3. American Diabetes Association Journals (Diabetes, Diabetes Care, Clinical Diabetes, Diabetes Spectrum)
4. Saudi Medical Journal, Omani Medical Journal, Kuwait Medical Journal, Bahrain Medical Bulletin, Qatar Medical Journal

**Describing the methods used to search relevant organisation sources**

1. Saudi Diabetes & Endocrine Association (<http://sdea.org.sa/>)
2. MENA Diabetes Leadership Forum 2010 Dubai (<https://www.novonordisk.com/content/dam/Denmark/HQ/aboutus/documents/MENA_Diabetes_briefing_book_EN.pdf>)
3. Ministry of Health Saudi Arabia Research Gate (<http://www.researchgate.net/institution/Ministry_of_Health_Saudi_Arabia>)
4. World Health Organization

(<http://search.who.int/search?q=SELF+MANAGEMENT+TYPE+2+DIABETES&spell=1&ie=utf8&site=who&client=_en_r&proxystylesheet=_en_r&output=xml_no_dtd&access=p&lr=lang_en>)

**Describing others searches included**

1. The reference lists of all records included in the review and relevant systematic reviews will be hand searched to identify additional records for inclusion.
