# Supplementary material for: Self-management of type 2 diabetes in gulf cooperation council countries: A systematic review
Source: PLoS One. 2017 Dec 12;12(12):e0189160. doi: 10.1371/journal.pone.0189160 (PMC5726716; doi:10.1371/journal.pone.0189160)
Supplement: S1 Table — (DOCX) [file pone.0189160.s002.docx]

**S 1 Table. Quality assessment**

| **Quality assessment** | **Authors** | Al-Daghri et al. 2014 [21] | Abduelkarem & Sackville. 2009 [22] | Mohamed et al. 2013 [19] | Al-Sinani et al. 2010 [25] | Al-Shahrani et al. 2012 [20] | Al Hayek et al. 2013 [24] | Al Asmary et al. 2013 [23] | Omer et al. 2015 [26] |
| --- | --- | --- | --- | --- | --- | --- | --- | --- | --- |
|  | **Yes 2,Partial 1, No 0, N/A not applicable** | | | | | | | | |
| 1. Question / objective sufficiently described? | | 2 | 2 | 2 | 2 | 2 | 2 | 2 | 1 |
| 2. Study design evident and appropriate? | | 1 | 0 | 2 | 1 | 2 | 2 | 2 | 1 |
| 3. Method of subject / comparison group selection or source of information/input variables described and appropriate? | | 1 | 0 | 2 | 2 | 2 | 2 | 2 | 1 |
| 4. Subject (and comparison group, if applicable) characteristics sufficiently described? | | 1 | 2 | 2 | 2 | 2 | 2 | 2 | 0 |
| 5. If interventional and random allocation was possible, was it described? | | N/A | N/A | 0 | N/A | N/A | N/A | N/A | N/A |
| 6. If interventional and blinding of investigators was possible, was it reported? | | N/A | N/A | 0 | N/A | N/A | N/A | N/A | N/A |
| 7. If interventional and blinding of subjects was possible, was it reported? | | N/A | N/A | 0 | N/A | N/A | N/A | N/A | N/A |
| 8. Outcome and (if applicable) exposure measure(s) well defined and robust to measurement / misclassification bias? Means of assessment reported? | | 2 | 1 | 2 | 2 | 2 | 1 | 2 | 1 |
| 9. Sample size appropriate? | | 2 | 1 | 2 | 2 | 2 | 2 | 0 | 2 |
| 10. Analytic methods described/justified and appropriate? | | 1 | 2 | 2 | 2 | 0 | 1 | 2 | 0 |
| 11. Some estimate of variance is reported for the main results? | | 2 | 2 | 2 | 2 | 2 | 0 | 2 | 0 |
| 12. Controlled for confounding? | | 0 | 0 | 2 | 0 | 0 | 0 | 0 | 0 |
| 13. Results reported in sufficient detail? | | 2 | 2 | 2 | 2 | 2 | 0 | 2 | 0 |
| 14. Conclusions supported by the results? | | 1 | 1 | 2 | 2 | 1 | 1 | 1 | 0 |
| **Summary score** | | 0.68 | 0.59 | 0.78 | 0.86 | 0.77 | 0.59 | 0.77 | 0.27 |
