# Supplementary material for: Self-management of type 2 diabetes in gulf cooperation council countries: A systematic review
Source: PLoS One. 2017 Dec 12;12(12):e0189160. doi: 10.1371/journal.pone.0189160 (PMC5726716; doi:10.1371/journal.pone.0189160)
Supplement: S2 Table — (DOCX) [file pone.0189160.s003.docx]

**S 2 Table. Explanation for coding category**

| **Codes** | **Subheadings** | **Description** |
| --- | --- | --- |
| **Education / Knowledge** | Dietary | Food habits correction and adopting healthy food knowledge are given to individuals. Interventionists work with participants together about a dietary program. |
|  | Physical Activity Guidance | A guideline about how physical activities to be done. |
|  | Other Sources | Sources are providing anything to improve self-management principles. |
| **Lifestyle** | Healthy Food | Incorporating nutritional management  into lifestyle |
|  | Being Active | Incorporating physical activity into  lifestyle |
|  | Monitoring | Monitoring blood glucose and other  parameters and interpreting and using  the results for self-management  decision making |
|  | Taking Medications | Using medication(s) safely and for  maximum therapeutic effectiveness |
| **Skills** | Problem Solving | Preventing, detecting, and treating acute  complications |
|  | Reducing Risks | Preventing detecting, and treating  chronic complications |
|  | Healthy Coping | Developing personalised strategies to  address psychosocial issues and  concerns |
| **Support** | Monitoring & Feedback | Support in the form of health monitoring and/or feedback on a regimen/promoted lifestyle change. |
|  | Psychological Interventions | Includes professional counselling or therapy for participants to provide psychological support. |
|  | Peer Support | It is provided by peers that refer to other patients who have diabetes type 2. This may be in the form of buddy system or through interaction with support groups. |
|  | Financial Incentives | The incentives are used to motivate participants to follow the steps in the whole process of the intervention. These aim to increase participants’ willingness levels to perform better and enhance their enthusiasm. |
